# A classification model for the telephonic triage of low acuity abdominal complaints in South Africa

**DOI:** 10.1016/j.afjem.2026.100999

**Published:** 2026-08-01

**Authors:** F Binks, LA Wallis, W Stassen

**Affiliations:** Division of Emergency Medicine, University of Cape Town, Cape Town, South Africa

**Keywords:** Triage, Emergency medical dispatch, Low acuity, Prehospital, CHAID

## Abstract

**Introduction:**

Emergency medical services (EMS) dispatch emergency resources according to the appropriate patient acuity, ensuring those in need of urgent care receive prompt attention. Abdominal complaints have been highlighted as an incident type that is over-triaged in EMS. The development of a telephonic classification tool could improve emergency medical dispatch efficiencies by reducing the inappropriate prioritisation of responses to these cases. The study aimed to identify variables that classified abdominal complaints as low acuity at dispatch.

**Methods:**

A retrospective cross-sectional study was performed on EMS data. Chi-square Automatic Interaction Detector (CHAID) was the classification model used in this study. Nodes split at p-values of 0.05, with the Bonferroni method used for correcting multiple comparisons. Classification tree validation used a 50/50 split and depth limits were applied to mitigate overfitting for training and testing samples.

**Results:**

Of the 14 inputs, four were significant predictors, producing a decision tree with 11 nodes. Mobility status was a strong predictor, with 93.3% of walking patients classified as low acuity. Apyrexia was more strongly associated with low acuity, and patients with no bleeding were more likely to be classified as low acuity than those with controlled bleeding. Specific age groups demonstrated a greater probability of being classified as low acuity relative to others. CHAID test model provided a predictive accuracy of 90.4%, a specificity of 50.00%, sensitivity of 92.10%, positive predictive value 94.55% and negative predictive value 40.18%.

**Conclusion:**

This study is the first of its type in a low- to middle-income country. It provides a potential classification system for low acuity abdominal complaints that may otherwise demand an emergent response from EMS. Applying these classification variables have the potential to inform and strengthen current emergency medical dispatch policies and processes. The dispatch algorithm can also be used to divert a low acuity case to more appropriate services.

## African relevance

• The Emergency Medical Dispatch (EMD) is the first point in the activation of the EMS system and is therefore critical in the dispatching of appropriate resources.
• Improved efficiencies in triaging of emergency calls in the EMD will improve resource utilization both in the prehospital and in-hospital environment.
• The EMD has the potential to influence the EMS system financially and economically which is required in a resource constrained environment.
• Over utilization of EMS resources is a global problem that has greater effects on the low to middle income countries.

## Introduction

Emergency Medical Services (EMS) are critical in providing prehospital care to patients needing urgent medical attention. Efficient EMS systems depend on the accurate prioritisation of emergency calls to ensure that the patient receives the correct EMS resource [1]. This responsibility lies with Emergency Medical Dispatch (EMD), which assesses the acuity of the patient telephonically and dispatches the most appropriate response [2,3]. Inappropriate dispatching of EMS resources can lead to over-triage, where non-emergent cases receive an emergency response [2,3]. Over-triage is a global issue, with prevalence rates ranging from 23.4% to 78% [[3], [4], [5], [6], [7], [8], [9], [10]].

Dispatching EMS resources to these lower acuity calls can drastically affect the efficiency of the EMS system by extending response times, limiting EMS and hospital resource availability, potentially compromising the ability of EMS to respond to life-threatening emergencies effectively and even delaying definitive care of patients with true emergencies [8,11].

South Africa has reported over-triage to be as high as 93.5% [12], with the Western Cape Government (WCG) EMS reporting 67.6% [10] and 62.2% in a more recent study [3]. These figures are much higher than the 35% recommendation made by the American College of Surgeons Committee on Trauma (ASCOT) [13]. Notably, these standards are derived for trauma and in a high-income country; as such, their direct transferability to other contexts or conditions is unclear. However, in the absence of other robust criteria, ASCOT has been used and referenced in low- to middle-income country (LMIC) studies globally as a benchmark for over- and under-triage [[14], [15], [16], [17]].

In the WCG, it is reported that 70% of emergency calls received are non-emergent, with at least 5000 callouts every month not requiring an EMS resource [18]. This is supported by a study in the same setting that reports 83% of patients receiving an emergency response received no medical intervention [15]. To address this issue, the development of dispatch tools specifically designed to identify low acuity cases is vital.

Abdominal complaints account for some of the most common low acuity hospital presentations in South Africa [19] and have the highest discharge rate of medical patients transported by EMS to hospital (34.4%) [20]. Studies have shown that abdominal complaints are among the most over-triaged conditions [2,3,10], with over-triage rates reported at 75% [3]. Abdominal complaints, when combined with vomiting and diarrhoea, also account for 11.74% of the total emergency case volume dispatched from the WCG EMD [3].

Given the prevalence of abdominal complaints, potential for incorrect telephonic triage, and impact on EMS resource utilisation [2,3,10,15,20,21], abdominal complaints represent a critical area for improving dispatch accuracy, potentially reducing over-triage. Therefore, this study aimed to identify specific variables that may classify abdominal complaints as low acuity in the dispatch of EMS resources.

## Methods

### Design

A retrospective cross-sectional study was conducted in South Africa's Western Cape Province by analysing the electronic patient care records (ePCR) used in EMS and the computer-aided dispatch (CAD) system used in the EMD. This study was approved by the Human Research Ethics Committee of the University of Cape Town (HREC Ref 547/2019).

### Setting

The Western Cape Department of Health manages the EMS in the Western Cape Province, which covers an area of 130,000 km² [22] and serves a population of 7.4 million people [23]. There are 269 ambulances with 1700 operational personnel which serviced 645,497 emergency cases in 2022/2023 [17].

The EMD receives all emergency calls and classifies them into two tiers (Priority 1 or P1 and Priority 2 or P2) based on information obtained from the caller's descriptions of signs and symptoms [2]. The P1 category is given higher priority and considered more urgent than the P2 category [2]. Once the call taker has received basic information about the emergency, an EMS resource is dispatched. All information regarding the case is documented in the CAD system in the EMD, and any case-related clinical information from the scene, patient treatment and management is recorded in the ePCR by the responding practitioners.

### Sample and sampling

This study collected data from the WCG for all primary response cases from 1 October 2018 to 30 September 2019 and excluded inter-facility transfer (IFT) of patients between medical facilities. This timeframe was intentionally selected to eliminate any confounding factors related to COVID-19 and lockdown measures. The data collected were also anonymised. The data files received from WCG were shared separately for CAD and ePCR through Microsoft Excel® (Microsoft Corp. Washington, United States) spreadsheets which excluded any patient identifiable information. The anticipated sample size was considered sufficient for CHAID analysis, which relies on large datasets to support iterative node splitting and to ensure stability and reliability of the resulting classification tree.

**Fig. 1:**
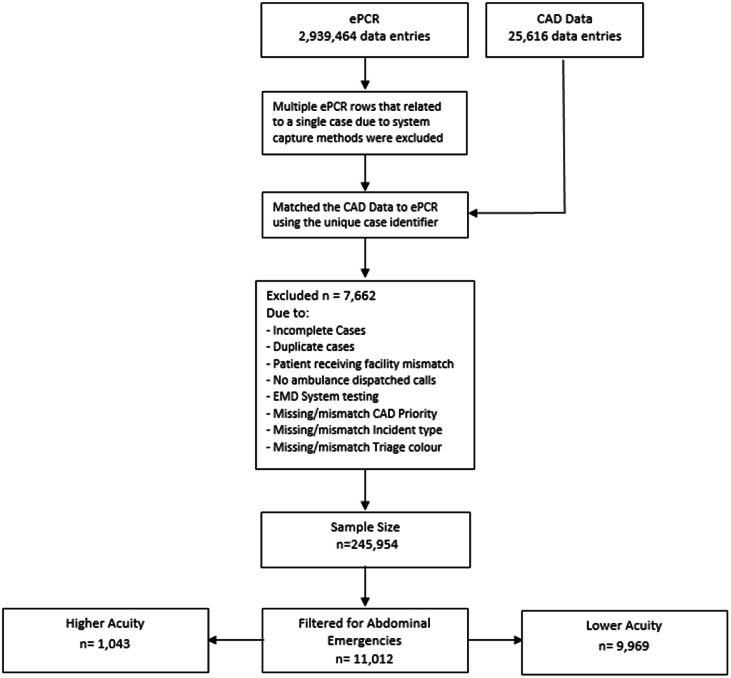
- Process flow of data.

### Data extraction and definitions

All cases received through the EMD were initially matched with their respective ePCR using unique case identifiers using STATA 17.0 (StataCorp., Texas, USA). It was noted that ePCR data included multiple entries per unique case identifier due to how information, vital signs, treatment and management are recorded. The ePCR cases with multiple entries per case were reviewed and reduced to a single entry using the last entry that relates to the specific case. Cases duplicated, mismatched, missing data, or cases containing system testing were excluded.

The cases were filtered for abdominal complaints only and classified into binary categories of lower and higher acuity. The term “abdominal complaints” is derived from the WCG EMD dataset and encompasses any medical concern related to the abdomen as reported by the caller during the emergency call . “Lower acuity” was defined as those cases that were triaged yellow or green according to the South African Triage Scale (SATS) [10]; while “higher acuity” was defined as those cases that were triaged as SATS red or orange [10]. This dichotomisation of the triage acuity was also used in other studies within the same setting [10,15], and suggested as a dependent variable for telephone triage system development [24].

### Statistical analysis and procedure

The data output in STATA 17.0 (StataCorp., Texas, USA) was then exported into IBM SPSS Statistics for Windows 29.0 (IBM Corp. Armonk, NY) for further analysis. Chi-square Automatic Interaction Detection (CHAID) was the model used to classify clinical indicators for abdominal complaints into cases of lower and higher acuity. Acuity was set as the dependent variable. No category of primary interest in CHAID was selected. As CHAID bases itself on statistical significance and associations between variables, selecting a category of primary interest may limit the algorithm’s ability to detect unanticipated associations between variables [25]. All independent variables were informed by established descriptors of low acuity through expert consensus [21]. CHAID determines the best split of variables starting at a root node, leading to a parent node, a child node and finally a terminal node, at which variables no longer split. This analytic approach has previously been suggested in the development of telephone triage systems [24].

Growth limits were set at a maximum tree depth of four, while the minimum number of cases for a parent node was set at n = 100 and a child node at n = 50. Merging categories and splitting nodes were set at p-values of 0.05. The Bonferroni method was applied to correct for multiple comparisons. Classification tree validation used a 50/50 split sample for training and test samples.

## Results

A total of 253,616 primary responses recorded in the EMD were matched to unique case identifiers on the ePCR. Following 7662 exclusions (Fig. 1), a sample size of 245,954 primary emergency responses was obtained. These cases were then filtered for abdominal complaints only, which yielded 11,012 cases eligible for inclusion and analysis, comprising 9969 (90.50%) lower acuity and 1043 (9.50%) higher acuity cases.

**Fig. 2:**
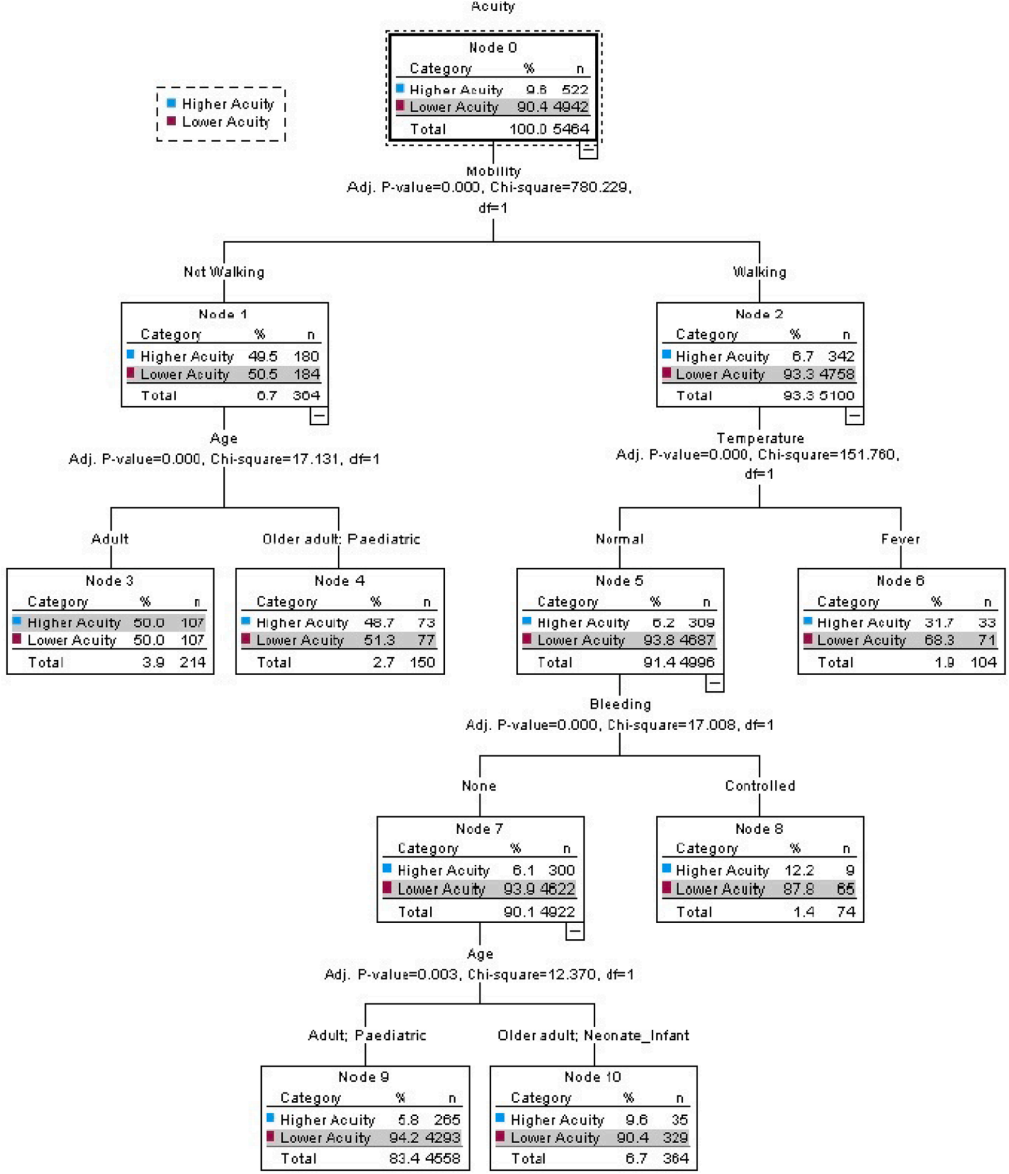
- CHAID test output results.

CHAID provided our independent variables of the 14 inputs with a total of 11 nodes (Fig. 2) and 6 terminal nodes. Predictors within each node are highlighted in the CHAID output result (Fig. 2). The parent node 0 indicates the dataset used in the test sample (n = 5464). The primary split in the classification tree was based on the patient's mobility (walking vs. not walking). Node 1 (not walking) is highlighted as being lower acuity at 50.5%. Subsequent results of node 1 within the tree also indicate approximate results of 50% between lower and higher acuity cases. Nodes 3 adult patients who are not walking are in favour of higher acuity.

Regarding node 2 (walking), the next variable was temperature, with patients without a fever favouring lower acuity at 94.3%. Walking patients with fever is a terminal node and resulted in lower acuity at 68.3%. The categories of no bleeding and controlled bleeding indicated lower acuity at 94.5% and 82.3%, respectively. Age categories associated with lower acuity ranged from 90.4% to 94.8%.

Table 1 shows the test sample classification and diagnostic accuracy of the data. The total cases in the training split were 5548 cases, and in the test split were 5464 cases. The model showed strong predictive performance for lower acuity cases with an accuracy of 97.8%, while only correctly predicting higher acuity cases at 20.5%. The CHAID model provided a high overall predictive accuracy of 90.4% within the test sample; however it is noted that lower-acuity cases comprised 90.4% of the dataset. This imbalance between high acuity and low acuity cases necessitated the inclusion of the F1 score (93.3%) as an additional classification metric for the lower acuity cases.

**Table 1:** CHAID classification and diagnostic test.

Test Sample Classification				
Sample	Observed	Predicted		
		Higher Acuity	Lower Acuity	% Correct
**Training**	Higher Acuity	132	389	25.3%
	Lower Acuity	99	4928	98.0%
	Overall Percentage	4.2%	95.8%	91.2%
**Test**	Higher Acuity	107	415	20.5%
	Lower Acuity	107	4835	97.8%
	Overall Percentage	3.9%	96.1%	90.4%

Test Sample Diagnostic Accuracy				
Statistic		Value	95% CI	
Sensitivity		92.10%	91.33% - 92.81%	
Specificity		50.00%	43.11% - 56.89%	
Lower acuity Prevalence		90.40%		
Positive Predictive Value		94.55%	93.81% - 95.20%	
Negative Predictive Value		40.18%	36.34% - 44.15%	
Positive Likelihood Ratio		1.84	1.61 - 2.11	
Negative Likelihood Ratio		0.16	0.13 - 0.19	
Pre-test odds		9.42		
Post-test odds LR+		17.33		
Post-test Probability LR+		94.5%	93.8% - 95.2%	
Post-test odds LR-		0.017		
Post-test Probability LR-		60.1%	55.5% - 64.6%	
Accuracy		88.05%	87.16% to 88.90%	
F1 score		93.3%		

## Discussion

This study aimed to identify the significant classification variables for low acuity abdominal complaints that can be used in the telephonic dispatch of emergency resources. Using CHAID, an algorithm structure of variables for the EMD to dispatch emergency resources to cases of low acuity abdominal complaints is provided.

The dataset was large enough, which supported a 50/50 split between training and test datasets. The training and test data reporting an accuracy of 91.2% and 90.4%, respectively, indicates that the algorithm in the model is not overfitted in providing classifiers in unseen data. It should be noted that the high prevalence of low acuity cases (90.4%) will influence the diagnostic accuracy. The reported PPV is therefore logically inflated at 94.55%. Despite the high prevalence of low acuity cases, sensitivity reported 92.10% indicating that most of the low acuity cases were correctly classified. These diagnostic accuracies support the credibility and reliability of CHAID in classifying low acuity abdominal complaints.

Over triage is reduced, providing confidence in dispatching resources appropriately for low acuity cases. However, with a specificity of 50.00%, CHAID correctly identified only half of the high acuity cases as not low acuity. With the high prevalence of low acuity and its inherent impact on diagnostic accuracy, likelihood ratios and post-test probabilities are calculated to offer more informative performance measures. This finding is further supported by the F1 score for low acuity. This misclassification poses some risk of under-triage and therefore highlights the importance of maintaining clinical judgment, additional review and prospective validation before operational implementation. Future classification models might need to be developed to optimise high acuity classification and then rationalised.

When CHAID classifies a case as low acuity, the actual probability of the case being low acuity rises from 90.4% (node 1) to 94.5%. This, combined with a positive likelihood ratio (LR+) of 1.84, signals strong confidence in confirming that a case is indeed low acuity. The negative likelihood ratio (LR−) of 0.16 and post-test probability LR- of 60.1% suggest a decreased certainty that a case is low acuity. Consequently, this may lead to under-triage, emphasising the importance of clinical judgement in reducing the risk of misclassification.

Considering that 90.4% of all abdominal complaints reported in this study are lower acuity (root node), it is surprising to note that these patients have an OR 0.58 (95% CI: 0.45–0.74; *p* < 0.01) of not being transported to the hospital [15]. This is reported despite being over-triaged [2] and having a high discharge rate from the hospital after EMS transport [20]. This discrepancy suggests that many patients were over triaged and may not have required EMS transport to the hospital. Such overuse of EMS resources can limit the availability of ambulances for genuine high acuity cases and potentially delay response times to these critical cases. Additionally, unnecessary transport contributes to the growing burden on emergency centres, exacerbating overcrowding and straining already limited healthcare resources [20].

Nodes 1 and 2 represent the initial split in the classification tree, distinguishing between cases where abdominal complaint classifiers are applicable to low-acuity dispatch decisions and those where they are not. Node 1, which includes patients who were ‘not walking’, along with its subsequent nodes, consistently performs around 50%. As such, this pathway is considered unreliable for supporting low acuity dispatch decisions. One node within this pathway was associated with higher acuity involving adult patients. These cases may involve more serious underlying conditions such as liver cirrhosis or cancer, which are known to be associated with higher rates of hospital admission and mortality in cases of abdominal pain [23]. These cases may also be viewed as patients with reduced mobility and having pre-existing conditions with a change in their chronic pain, placing them in a category of higher acuity [21].

The nodes after node 2 provide the algorithm for classifying the dispatch of abdominal complaints to cases of lower acuity. The walking patient in node 2 is identified as low acuity at 93.3%, and patient temperature is identified as the next descriptor. With fever being a terminal node reporting low acuity at 68.3%, although being low acuity, it is a terminal node and not advisable to include this result as a classifier for the algorithm. This descriptor can be beneficial in identifying cases of sepsis, with temperature being one of the key descriptors used in screening tools, such as the Robson Prehospital Severe Sepsis Screening tool [26]. South African EMS has been shown to be moderately accurate in recognising sepsis [27] and EMS recognition with early treatment has a substantial impact on patient outcomes [28,29]. It is noted that other key descriptors of sepsis include gastrointestinal keywords that are associated with abdominal complaints [30]. Combining these gastrointestinal keywords and temperature makes up 52% of the descriptors used in the telephonic identification of sepsis [30] in the EMD. At this point of the classification tree, it may be beneficial to introduce alternative branches and expand the dispatch algorithm to explore cases with possible sepsis. It is recommended that the evidenced keywords and descriptors for sepsis [30] be incorporated into a telephonic dispatch algorithm to support the development of alternative branches within the CHAID classification tree.

The classification tree establishes that a patient walking with no fever may be regarded as a low acuity patient. Obtaining information telephonically about a patient having a fever or not may have been challenging previously; however, recent developments may have improved the feasibility and reliability of this assessment. It can be argued that the COVID-19 pandemic has heightened awareness and knowledge about temperature monitoring. The widespread implementation of temperature screening has increased people's familiarity with temperature measurement techniques and their understanding of the importance of fever in identifying potential illness [31]. This temperature awareness, coupled with the dichotomy of the question, supports including this descriptor in the low-acuity dispatch tool.

According to expert consensus, a low acuity abdominal complaint case must have no bleeding through any orifice [21]. Although CHAID highlighted lower acuity for controlled bleeding at 87.8%, the frequencies of these calls are only 1.4% of the total volume. Telephonic confirmation of bleeding or identification of melena may also prove difficult. EMS may use discretion in their classification of including this variable as part of the low acuity dispatch tool.

While the decision tree does not classify any age group as high acuity, caution is advised for paediatric patients. Rotavirus is responsible for 70% of paediatric gastroenteritis cases and presents a significant risk of dehydration and mortality [32]. Advancements in water sanitation, personal hygiene, and rotavirus vaccines have significantly reduced diarrhoeal mortality rates in South Africa over the past three decades [32,33]. Despite the results of low acuity in these age categories being convincing, incorporating these descriptors into the low acuity dispatch tool may depend on the risk tolerance of the EMS operation.

**Fig. 3:**
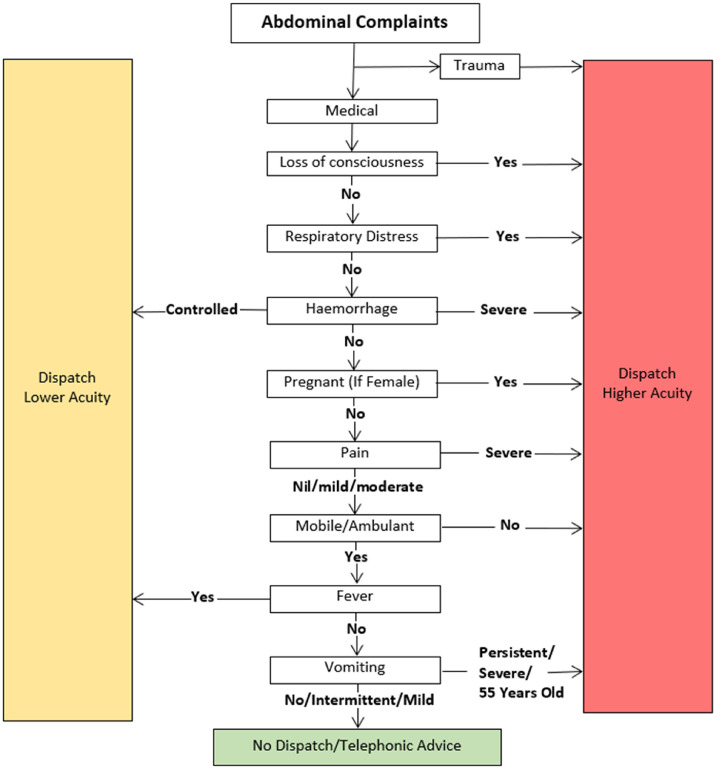
Schematic diagram of low acuity abdominal complaint dispatch tool.

In dispatching EMS to cases with abdominal complaints, three descriptors must be first ruled out: 1) the patient must be conscious, 2) there must be no respiratory distress, 3) there must be no signs of severe haemorrhage [21]. Thereafter, CHAID as a classification tree has provided statistically significant classifiers to be used in an algorithm for the low acuity dispatch of abdominal complaints. The practical application and integration of these classifiers into dispatch decision-making is illustrated in Fig. 3.

The low acuity dispatch classification tree informed by expert consensus [21], is a substantial stride in addressing the issues of over-triage and inappropriate response to such cases. The methodology could be replicated for other chief complaints. However, some refinement and validation through pilot testing is warranted.

### Limitations

This study was conducted using data from the Western Cape, South Africa and may not be valid to use elsewhere. It is recommended that this study be replicated in other settings to obtain results specific and applicable to that setting. Data in this study were obtained from ePCR and CAD data. Information may have been incorrectly entered by EMS personnel; however, this likely would not be substantial enough to influence the outcome of the study due to the sample size used. Although CHAID detects interactions between variables in the study, it did not capture all the variables, such as high-risk pregnancy or menstruation, as these variables may have been low in prevalence and not statistically significant. CHAID may also lose the complexities between binary classifications, such as patient walking vs not walking, where factors such as pain or fatigue may have influence on a patient’s ability to mobilise. As this model is intended to support clinical dispatch decision-making, future prospective validation studies should assess model calibration and observed clinical outcomes across external populations.

## Conclusion

This study, being the first of its type in LMICs, provides a tool for the telephonic dispatch of EMS resources for low acuity abdominal complaint cases. The findings provide a foundational proof of concept for the development of a dispatch support tool that may assist the EMD in identifying cases that could potentially be managed through alternative pathways, such as private transportation, clinical advice, or non-emergent responses. Further validation and testing in various operational EMD environments, CAD or criteria-based dispatch systems should be undertaken before deployment.

## CReDiT author contributions

FB: Conceptualisation, Methodology, Formal Analysis, Investigation, Writing: Original Draft,Project administration; LW: Methodology, Writing: Review and Editing, Validation, Supervision; WS: Conceptualisation, Methodology, Validation, Writing: Review and Editing, Supervision, Funding acquisition.

## Funding

This study was partly funded through a National Research Foundation of South Africa grant (Thuthuka Grant Nr 121,971), held by WS.

## Dissemination of results

The results of this study were shared with the Western Cape Government.

## Declaration of competing interest

WS is an Associate Editor for this journal but was not involved in the editorial review or the decisions to publish this article.
